# Vitamin D and the metabolic-associated steatotic liver disease—type 2 diabetes axis: a scoping-narrative review of global evidence and emerging perspectives for Sub-Saharan Africa

**DOI:** 10.3389/fepid.2026.1765215

**Published:** 2026-03-11

**Authors:** Bruno Basil

**Affiliations:** International Institute of Pathology and Forensic Science Research, David Umahi Federal University of Health Sciences, Uburu, Nigeria

**Keywords:** metabolic syndrome, metabolic-associated steatotic liver disease (MASLD), Sub-Saharan Africa, type 2 diabetes mellitus, Vitamin D, Vitamin D paradox

## Abstract

**Background:**

Metabolic dysfunction-associated steatotic liver disease (MASLD) and Type 2 Diabetes Mellitus (T2DM) are rapidly emerging as twin epidemics in Sub-Saharan Africa (SSA), driven by urbanization and nutritional transition. While global evidence links Vitamin D deficiency (VDD) to the progression of both disorders, data specific to African populations remains fragmented. This review explores the Vitamin D–MASLD–T2DM axis, contrasting global mechanistic insights with the unique genetic, environmental, and infectious disease landscape of SSA.

**Methods:**

A hybrid scoping-narrative review was conducted searching PubMed/MEDLINE, Scopus, and Embase for literature published up to 2025. The search targeted mechanistic studies, clinical trials, and regional epidemiological data. Out of 948 initial citations, 59 high-quality studies were prioritized for synthesis. The review integrates molecular evidence of Vitamin D Receptor (VDR) signaling with clinical outcomes and evaluates their applicability to the African context.

**Results:**

Mechanistic evidence indicates that Vitamin D exerts potent anti-inflammatory and insulin-sensitizing effects via VDR activation, specifically by downregulating hepatic *de novo* lipogenesis (SREBP-1c) and suppressing NF-κB signaling in Kupffer cells. Epidemiological data consistently associate VDD with increased liver fibrosis and insulin resistance. However, randomized controlled trials yield conflicting results, likely due to heterogeneity in dosing and baseline status. Uniquely in SSA, the “Vitamin D Paradox” (low total levels with preserved bone health), the rarity of the *PNPLA3* genetic risk variant, and the metabolic toxicity of antiretroviral therapy (e.g., Efavirenz) create a distinct pathophysiological environment where standard definitions of deficiency may be inadequate.

**Conclusion:**

Vitamin D deficiency is a plausible, modifiable driver of the MASLD–T2DM axis in Sub-Saharan Africa, potentially filling the risk void left by the absence of major genetic drivers like *PNPLA3*. However, Eurocentric thresholds for deficiency may not apply. Future research must prioritize establishing ancestry-specific reference ranges and conducting region-specific trials that account for the “triple burden” of HIV, urbanization, and dietary transition to inform effective public health interventions such as fortification.

## 1 Introduction

Current metabolic health trends reveal a steady global rise in chronic diseases fuelled by overnutrition and sedentary lifestyles. Metabolic dysfunction-associated steatotic liver disease (MASLD) has paralleled the global obesity epidemic to become the most common cause of chronic liver disease worldwide, affecting an estimated 30% of the adult population (1, 2). It represents a spectrum of disease severity, ranging from simple steatosis (accumulation of triglycerides in >5% of hepatocytes) to metabolic dysfunction-associated steatohepatitis (MASH), which is characterized by hepatocyte injury (ballooning), inflammation, and varying degrees of fibrosis (2, 3). Concurrently, Type 2 Diabetes Mellitus (T2DM) has reached pandemic proportions, with the International Diabetes Federation (IDF) projecting that the number of adults living with diabetes will increase by 51% globally by 2045 (4). The relationship between MASLD and T2DM is causal and bidirectional. Evidence shows that T2DM is an independent risk factor for the progression of simple steatosis to MASH and cirrhosis, doubling the risk of hepatocellular carcinoma even in the absence of cirrhosis (5). Conversely, the liver plays a central role in glucose homeostasis, and hepatic insulin resistance is a primary driver of fasting hyperglycaemia in T2DM (6). These two disorders create a self-perpetuating cycle that contributes to worsening metabolic dysfunction at the population level.

Sub-Saharan Africa (SSA) represents a metabolic setting that is distinct from other world regions because of its rapid socioeconomic transition, evolving dietary patterns, and complex interaction between undernutrition and emerging overnutrition. The region is undergoing a nutrition transition in which traditional diets rich in legumes, whole grains, and vegetables are increasingly being replaced by refined carbohydrates, processed foods, sugary beverages, and high-fat meals (7). This shift, driven by urbanization and globalization, has produced a “double burden of malnutrition” where stunting and micronutrient deficiencies coexist alongside rising rates of overweight and obesity (8). Despite the rising prevalence of metabolic disease, data on MASLD in SSA remains sparse compared to Western regions. Recent estimates suggest a pooled prevalence of approximately 29.21%, with rates as high as 46% in obese sub-populations (9). However, the clinical presentation and genetic drivers may differ. For instance, the *PNPLA3* I148M variant (rs738409 C > G), the strongest genetic risk factor for MASLD in Europeans and Hispanics, is rare in African populations, suggesting that environmental factors or other genetic loci play a more dominant role (10).

In the search for modifiable risk factors connecting these two epidemics, Vitamin D has emerged as a biologically plausible modulator because of its widespread actions across metabolic tissues. Although traditionally known for its regulation of calcium and bone metabolism, the discovery of the Vitamin D Receptor (VDR) in nearly all human tissues including the liver, pancreas, adipose tissue, and skeletal muscle, has unveiled its role as a potent pleiotropic hormone (11). The active metabolite, 1,25-dihydroxyvitamin D, functions as a transcription factor regulator. In the pancreas, it is essential for normal insulin secretion and beta-cell survival (11), while in the liver and adipose tissue, it modulates inflammation and insulin sensitivity (12). Global epidemiological data consistently show an inverse association between serum 25-hydroxyvitamin D [25(OH)D] levels and the risk of developing both T2DM and MASLD (13–15). In the context of SSA, Vitamin D is particularly relevant because sunlight exposure is abundant yet paradoxical deficiency remains common due to skin pigmentation, urban indoor lifestyles, and nutritional inadequacies (16, 17). These biological and environmental factors make Vitamin D a compelling candidate modifier of the MASLD and T2DM relationship and a potentially important factor in understanding the drivers of metabolic disease in SSA.

Despite strong global evidence linking Vitamin D to the MASLD and T2DM pathway, a substantial knowledge deficit exists regarding how this relationship manifests within SSA. Current understanding is derived almost entirely from Caucasian, Asian, and African American populations (18), which limits its relevance because SSA populations have distinct biological, environmental, and epidemiological characteristics. Genetic variations affecting Vitamin D Binding Protein and the VDR alter Vitamin D transport, bioavailability, and downstream signaling in ways that may differ from other populations (19). Environmental exposures also create a unique metabolic milieu, while high HIV prevalence and widespread use of antiretroviral therapy introduce metabolic alterations that are not seen in most global cohorts (20). Additionally, prevailing definitions of Vitamin D deficiency (VDD) were established using bone health criteria in Western populations and may not accurately reflect thresholds associated with metabolic risk in Africans (21). All these factors make direct extrapolation of international findings to SSA both challenging and potentially misleading.

This review aims to address this critical gap by synthesizing global mechanistic and clinical insights alongside the limited but growing body of evidence in SSA. By examining how genetic diversity, environmental pressures, infectious disease burdens, and evolving dietary patterns interact with Vitamin D biology in SSA, this study seeks to clarify how Vitamin D may influence the relationship between MASLD and T2DM in this region. The main goal is to provide a structured roadmap that identifies priority research questions, highlights regionally relevant determinants, and evaluates the potential for Vitamin D to serve as a modifiable target in the prevention and management of metabolic diseases like MASLD and T2DM across SSA.

## 2 Methods

To achieve a comprehensive analysis that captures both established global evidence and the unique characteristics of the Sub-Saharan African context, this report adopts a hybrid methodological approach.

### 2.1 Study design

The study was structured as a scoping review adhering to the methodological stages outlined by Arksey and O'Malley which includes: (1) identifying the research question, (2) identifying relevant studies, (3) study selection, (4) charting the data, and (5) collating, summarizing, and reporting the results (22). This approach supported the inclusion of a wide range of evidence, such as mechanistic studies, clinical research, and region-specific data from SSA, allowing the review to map key concepts, evidence gaps, and the breadth of available literature. In addition to the scoping methodology, a narrative interpretive component was incorporated to integrate mechanistic pathways, clarify the MASLD–T2DM axis, and develop emerging perspectives relevant to the SSA context.

### 2.2 Search strategy

A systematic search was executed across primary bibliographic databases including PubMed/MEDLINE, Scopus, Web of Science, and Embase. To ensure high sensitivity, the search strategy employed a Boolean logic combining Medical Subject Headings (MeSH) and free-text terms organized into three distinct clusters:

- Cluster 1 (Metabolic Disease): “Metabolic dysfunction-associated steatotic liver disease” OR “MASLD” OR “MAFLD” OR “Non-alcoholic fatty liver disease” OR “NAFLD” OR “NASH” OR “Steatohepatitis” OR “Type 2 Diabetes” OR “T2DM” OR “Insulin Resistance” OR “Metabolic Syndrome”.
- Cluster 2 (Vitamin D): “Vitamin D” OR “Cholecalciferol” OR “Ergocalciferol” OR “25-hydroxyvitamin D” OR “Calcitriol” OR “Vitamin D Deficiency” OR “Vitamin D Receptor” OR “VDR”.
- Cluster 3 (Population/Region): “Sub-Saharan Africa” OR “Africa” OR “West Africa” OR “East Africa” OR “South Africa” OR “Nigeria” OR “Kenya” OR “Ghana” OR “African ancestry” OR “Black population”.

In addition to the database search, reference lists of included articles were hand-searched, and forward citation tracking was performed to identify recent studies that may not yet be fully indexed.

### 2.3 Selection strategy

The selection of studies followed clearly defined inclusion and exclusion criteria to ensure relevance and rigor. Studies were included if they involved adult human populations aged 18 years or older, while paediatric cohorts were considered only when addressing the Developmental Origins of Health and Disease (DOHaD) hypothesis. Eligible studies examined the association, mechanistic link, or therapeutic effect of Vitamin D on MASLD, T2DM, or related intermediate metabolic markers, such as HOMA-IR, liver enzyme levels, or fibrosis scores. Although global studies were included to provide mechanistic insights, priority was given to research conducted in Sub-Saharan Africa or involving African diaspora populations to enhance regional applicability. Eligible study designs comprised observational studies, including cross-sectional, case-control, and cohort studies, as well as randomized controlled trials, systematic reviews, and meta-analyses.

Studies were excluded if they focused solely on Type 1 Diabetes or alcohol-associated liver disease, involved animal models unless used to elucidate mechanisms not yet demonstrated in humans, or were published in languages other than English. This structured approach ensured that the review captured both the breadth of global evidence and the specificity of the SSA context.

### 2.4 Data extraction

Data extraction was structured to populate three analytical streams. The first stream focused on global mechanistic evidence, examining pathways linking Vitamin D receptor activation to hepatic lipid metabolism, insulin signaling, and inflammatory processes. The second stream captured global clinical evidence, including epidemiological associations and outcomes from randomized controlled trials related to Vitamin D status and MASLD or T2DM progression. The third stream emphasized SSA-specific contextual evidence, encompassing prevalence data, genetic associations (VDR, DBP, PNPLA3), dietary patterns, and the metabolic effects of HIV and antiretroviral therapy.

### 2.5 Synthesis approach

Given the heterogeneity of included studies, which ranged from molecular biology investigations to broad epidemiological surveys, a meta-analysis was not feasible. Instead, a narrative synthesis was employed to integrate quantitative data (e.g., prevalence rates) with qualitative insights (e.g., hypotheses regarding the “Vitamin D Paradox”). The synthesis was organized to first establish universal biological plausibility and then assess how these mechanisms apply within the specific environmental, genetic, and epidemiological realities of SSA.

### 2.6 Limitations

The primary limitation of this review reflects the broader field. High-quality, biopsy-confirmed MASLD studies are scarce in SSA, and regional studies often rely on liver enzymes or ultrasound, which have limited sensitivity for mild steatosis and fibrosis, potentially leading to an underestimation of the true disease burden. Furthermore, available data are predominantly derived from urban and hospital-based cohorts, thereby limiting generalizability to rural populations where lifestyle and environmental exposures differ. Methodological limitations in the included observational studies also introduce the possibility of residual confounding by adiposity, lifestyle factors, and socioeconomic status, which were not uniformly controlled for across the literature. Additionally, variations in the definition of VDD across studies complicate direct comparisons between global and SSA cohorts.

## 3 Results

### 3.1 Search results and study characteristics

**Figure 1:**
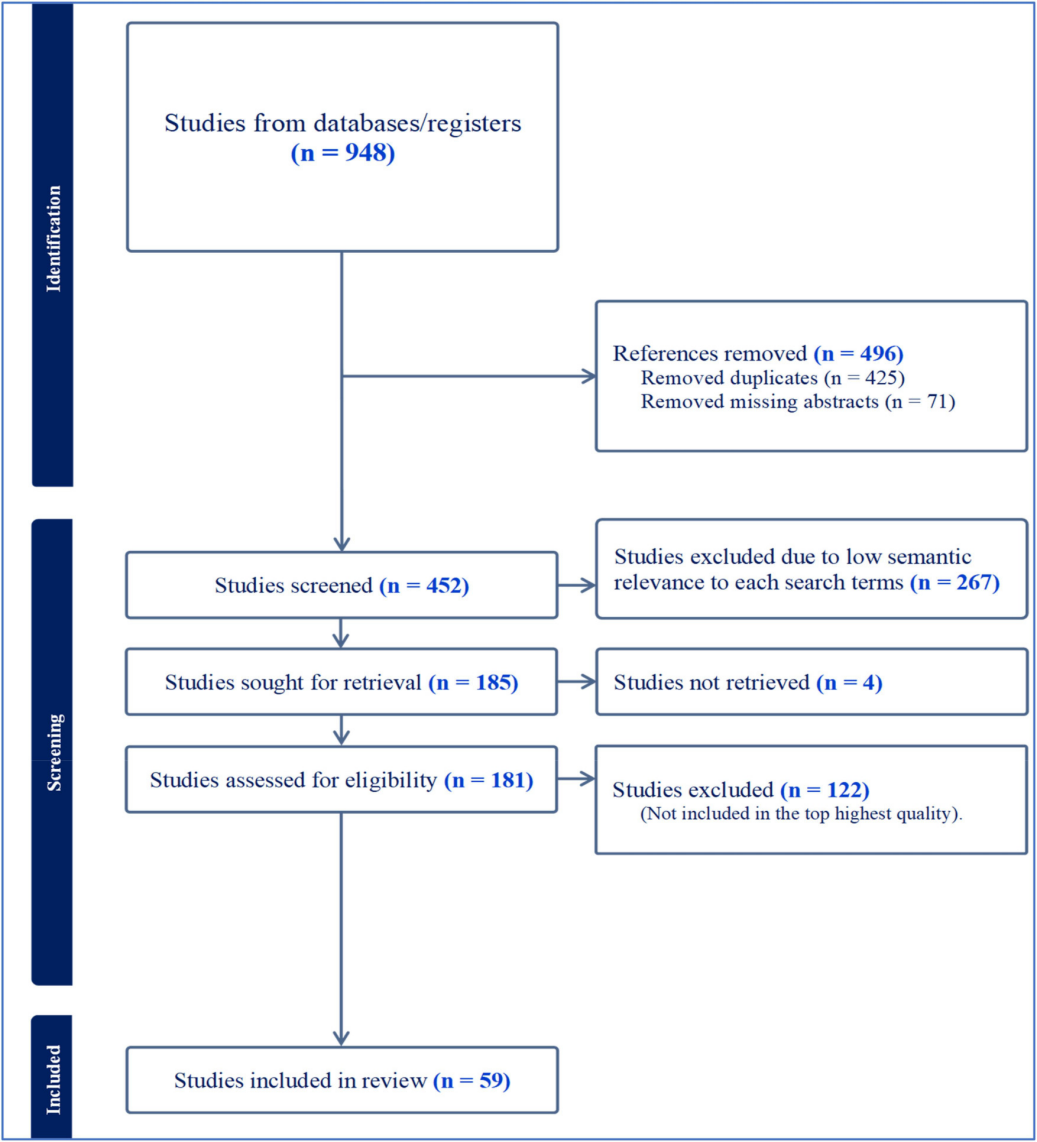
PRISMA flow diagram illustrating the literature search and selection process.

The systematic search strategy yielded a total of 948 citations. Following the removal of duplicates and articles with missing abstracts, 452 unique citations remained for screening. After reviewing titles and abstracts for relevance to the Vitamin D–MASLD–T2DM axis, 181 full-text articles were assessed for eligibility. Ultimately, 59 high-quality studies were prioritized for the final synthesis. These included global mechanistic reviews, clinical trials, and region-specific observational studies from Sub-Saharan Africa. The selection process is detailed in Figure 1.

### 3.2 Global perspectives on the Vitamin D–MASLD–T2DM relationship

#### 3.2.1 Vitamin D and MASLD—mechanistic and epidemiological links

The liver is central to Vitamin D metabolism, converting cutaneous or dietary Vitamin D3 into 25(OH)D via the enzyme CYP2R1. However, hepatocytes and non-parenchymal liver cells also express the VDR, making the liver a target organ for Vitamin D action (23, 24) Experimental models demonstrate that VDR activation suppresses *de novo* lipogenesis. It achieves this by downregulating the expression of Sterol Regulatory Element-Binding Protein 1c (SREBP-1c), a key transcription factor that drives the synthesis of fatty acids and triglycerides in the liver (12). Simultaneously, Vitamin D promotes hepatic beta-oxidation of fatty acids and enhances the secretion of Very Low-Density Lipoprotein (VLDL), thereby reducing intrahepatic lipid accumulation (11, 25). Also, chronic low-grade inflammation drives the progression from simple steatosis to MASH, and Vitamin D exerts potent anti-inflammatory effects by inhibiting the Nuclear Factor kappa B (NF-κB) signaling pathway in Kupffer cells (26). This suppression reduces the production of pro-inflammatory cytokines such as Tumour Necrosis Factor-alpha (TNF-α), Interleukin-6 (IL-6), and Interleukin-1 beta (IL-1β), which are implicated in hepatocellular injury and insulin resistance (27).

Epidemiological evidence reinforces these mechanistic insights. Several observational studies have shown that low Vitamin D levels are consistently associated with greater liver disease burden. Meta-analytic data in people with T2DM indicate that VDD more than doubles the risk of MASLD compared to individuals with adequate levels, and this relationship persists after accounting for common confounders such as age, sex, and adiposity. The association appears notably stronger in individuals with a BMI >23 kg/m² (28). Importantly, the severity of VDD tracks closely with histologic features of liver injury. Furthermore, lower serum 25(OH)D levels correlate with greater histological severity of NAFLD, including higher NAFLD Activity Scores (NAS) and advanced fibrosis stages (23).

#### 3.2.2 Vitamin D and T2DM—pathways of glucose regulation

Vitamin D plays a central role in pancreatic beta-cell physiology by supporting both insulin biosynthesis and secretion. Pancreatic beta-cells possess both VDR and the 1-alpha-hydroxylase enzyme (CYP27B1), allowing them to locally synthesize active 1,25(OH)2D (29). Vitamin D is essential for the biosynthesis of insulin and facilitates the calcium influx required for insulin exocytosis (11). Deficiency leads to impaired glucose-stimulated insulin secretion (GSIS) and may accelerate beta-cell apoptosis, a hallmark of progressive T2DM (29).

Vitamin D also acts on peripheral insulin-sensitive tissues, particularly skeletal muscle and adipose tissue, where it enhances insulin signaling through multiple pathways. It directly upregulates the expression of Insulin Receptor Substrate (IRS) proteins and facilitates the translocation of GLUT4 glucose transporters to the cell surface in skeletal muscle and adipose tissue (11, 30, 31). At the systemic level, Vitamin D suppresses systemic inflammation (lowering CRP, TNF-α), and reduces the “metabolic noise” that interferes with insulin signaling pathways (32). It additionally influences adipokine secretion, increasing levels of adiponectin (an insulin-sensitizing hormone) and decreasing leptin, thereby improving the metabolic profile of adipose tissue (11, 33). Through these combined actions, Vitamin D contributes to improved insulin sensitivity and a more metabolically resilient phenotype. These tissue-specific molecular mechanisms are summarized in Table 1.

**Table 1:** Comparative mechanisms of Vitamin D in the MASLD–T2DM axis.

Target Tissue	Molecular Mechanism (VDR Activation)	Physiological Outcome	Relevance to MASLD/T2DM Pathophysiology
Hepatocyte (11, 12, 23)	↓ SREBP-1c expression;	Reduced *de novo* lipogenesis	Prevents Steatosis (Liver Fat Accumulation) and lipotoxicity
	↑ Beta-oxidation enzymes	Enhanced VLDL secretion	
Kupffer Cell (12)	↓ NF-κB signaling;	Reduced cytokine release (TNF-α, IL-6)	Prevents progression to MASH (Inflammation)
	↓ TLR expression		
Hepatic Stellate Cell (11, 12, 23)	↓ TGF-β/SMAD signaling	Reduced myofibroblast activation and collagen deposition	Prevents Liver Fibrosis
Pancreatic Beta-Cell (11, 34)	↑ Insulin gene transcription	Improved Insulin Biosynthesis & Secretion; Reduced Apoptosis	Prevents Beta-cell failure (T2DM onset)
	Regulates Ca2 + flux		
Skeletal Muscle (30)	↑ IRS-1 expression;	Increased Glucose Uptake	Improves peripheral insulin sensitivity
	↑ GLUT4 translocation		
Adipose Tissue (33)	↑ Adiponectin secretion;	Improved Insulin Sensitivity	Reduces peripheral lipotoxicity (FFA flux to liver)
	↓ Leptin secretion	Reduced systemic inflammation	
Gut Microbiome (35, 36)	↑ Tight Junction proteins (Claudins)	Improved Gut Barrier Function	Reduces Metabolic Endotoxemia (LPS translocation)
	Modulates flora composition		

#### 3.2.3 Vitamin D—modulator of the MASLD–T2DM interaction

The bidirectional relationship between MASLD and T2DM creates a “vicious cycle” of metabolic dysfunction. Hepatic insulin resistance (driven by liver fat) results in unchecked gluconeogenesis and fasting hyperglycaemia. Simultaneously, peripheral insulin resistance increases the flux of free fatty acids to the liver, fuelling further steatosis. Within this cycle, VDD may act as a crucial “second hit” or disease modifier in this axis. In patients with established T2DM, deficiency exacerbates hepatic lipid deposition, accelerating the transition to MASH (28). Conversely, in patients with primary MASLD, deficiency impairs pancreatic compensation, hastening the onset of overt diabetes (34).

Although the mechanistic and observational data are compelling, results from Randomized Controlled Trials (RCTs) of Vitamin D supplementation have been inconsistent. Some meta-analyses indicate that Vitamin D supplementation significantly reduces HOMA-IR (a marker of insulin resistance), lowers ALT/AST levels, and decreases markers of oxidative stress (like malondialdehyde—MDA) and inflammation (hs-CRP) in patients with metabolic syndrome and MASLD (32). Other studies, including large trials, have found no significant effect on HbA1c, liver histology (steatosis or fibrosis), or mortality (14, 37, 38). These conflicting findings are likely due to heterogeneity in study designs. Most trials often fail to stratify by baseline Vitamin D status (supplementation is unlikely to help those who are already sufficient) or genetic variants. Furthermore, the dose and duration of supplementation vary widely, with daily physiological doses often showing different effects compared to massive bolus doses (32).

### 3.3 Evidence gaps in Sub-Saharan Africa

The global evidence provides a robust theoretical framework, but its application to Sub-Saharan Africa is fraught with uncertainty. The region exhibits specific epidemiological, genetic, and environmental features that may fundamentally alter the Vitamin D–MASLD–T2DM relationship.

#### 3.3.1 The epidemiology of the “triple burden” in SSA

There is a common misconception that VDD is rare in Africa because of abundant sunshine, but empirical data contradicts this. Systematic reviews estimate the prevalence of deficiency (<30 nmol/L) in SSA general populations to be approximately 18%–20%, with rates rising to over 50% in specific vulnerable groups such as women, newborns, and the elderly. It is also significantly more common in urban areas compared to rural ones, likely due to indoor lifestyles and pollution (16). In clinical cohorts of patients with type 2 diabetes in Nigeria and other African settings, studies have reported VDD rates ranging from 38% to 63% (39, 40). These rates are alarming given the established link between the vitamin deficiency and poor glycaemic control.

Recent meta-analyses place the pooled prevalence of MASLD in SSA at 29.2%, with important regional and demographic variation that helps explain the distribution of risk. West Africa shows the highest prevalence at 34.4%, a trend that may reflect rapid urbanization and dietary transitions characterized by increased consumption of palm oil and refined carbohydrates, while Southern Africa follows at 26.9%, where Westernized dietary patterns are more firmly established (9). An additional feature of the SSA epidemiology is the gender disparity. Unlike many Western cohorts where men show higher prevalence, MASLD in SSA is notably more common in women, with estimates of 27.1% in females compared with 23.0% in males (9, 41), a pattern that may relate to cultural perceptions of body size, higher obesity rates among women, and possibly hormonal factors that could interact with vitamin D status.

Furthermore, the explosion of T2DM in SSA is well documented, but the phenotype often differs from that observed in Western populations. Many African patients develop diabetes at a relatively low BMI, a pattern often referred to as “lean diabetes,” which suggests that visceral and ectopic fat deposition in organs such as the liver and pancreas may play a disproportionately large role compared with subcutaneous fat (42). Body composition studies in African populations indicate that even in the absence of overt obesity, ectopic fat accumulation contributes to insulin resistance, beta-cell stress, and metabolic dysregulation. This phenotype shares features with the lipodystrophy-like pattern seen in HIV-infected and treated individuals in SSA, where subcutaneous fat storage capacity is impaired, leading to redistribution of fat to visceral and ectopic depots, which further exacerbates insulin resistance and organ-specific metabolic dysfunction (43). The combination of lean diabetes, high rates of visceral and ectopic fat, and HIV-related fat redistribution highlights the unique metabolic context of T2DM in SSA, which differs significantly from classical obesity-driven diabetes patterns observed elsewhere.

#### 3.3.2 The “Vitamin D paradox” and genetic bioavailability

A critical scientific gap is the definition of “deficiency” itself. The “Vitamin D Paradox” refers to the observation that individuals of African ancestry consistently present with lower total serum 25(OH)D levels than Caucasians, yet paradoxically have superior bone mineral density and lower fracture rates (19, 44) In this context, Vitamin D Binding Protein (DBP) polymorphisms become particularly important. Approximately 85–90 percent of circulating Vitamin D is bound to DBP (GC globulin), while 10–15 percent is bound to albumin, and less than 1 percent is “free.” Moreover, African populations predominantly carry the Gc1f allele of the DBP gene, whereas Caucasians carry the Gc1s or Gc2 alleles (19). Early studies suggested that the African DBP variant had a lower affinity for Vitamin D, resulting in a higher percentage of free (bioavailable) hormone despite lower total levels, a concept referred to as the “Bioavailability Hypothesis,” and this would explain the bone health paradox (45).

However, more recent studies using direct measurement assays have challenged this, finding that free Vitamin D levels track with total levels and are indeed lower in Africans (21). This implies that the “deficiency” is real and that physiological adaptation, such as PTH resistance, or other mechanisms may be preserving bone. Consequently, if African tissues are adapted to lower intracellular Vitamin D levels, standard “insufficiency” cut-offs (e.g., <30 ng/mL) might be inappropriate for assessing metabolic risk; conversely, if the free hormone is truly low, then the widespread deficiency is a genuine emergency driving the metabolic epidemic, and this remains a massive research gap.

Furthermore, VDR polymorphisms add another layer of complexity. Genetic variations in the VDR gene (FokI, BsmI, TaqI) modulate receptor sensitivity, and the frequency of these alleles differs significantly in African populations. For example, the TaqI polymorphism has been associated with Vitamin D insufficiency in South African populations, while FokI has been linked to cancer risk in African Americans (46). The specific impact of these variants on hepatic lipid metabolism in Africans is unknown, and this represents an additional gap requiring focused investigation.

#### 3.3.3 The “protective” genetic profile—PNPLA3

In European and Hispanic populations, the PNPLA3 I148M variant (G allele) is the single strongest genetic predictor of MASLD, driving liver fat accumulation even in the absence of obesity. However, the African anomaly becomes immediately apparent, as this risk allele is rare in African populations (allele frequency ∼19 percent vs. ∼49 percent in Hispanics) (47). Yet the paradox is that despite possessing a “protective” genetic profile regarding PNPLA3, SSA populations are experiencing a surge in MASLD. Taken together, this strongly suggests that environmental factors, including diet and physical activity, as well as other genetic modifiers, are driving the epidemic in SSA. Consequently, it creates a compelling niche for VDD to act as a primary driver, because in the absence of strong genetic susceptibility such as PNPLA3, environmental insults like VDD may have a higher attributable risk fraction for MASLD in Africans than in other groups (48).

#### 3.3.4 The confounding role of HIV/ART

The intersection of viral infection, treatment, and metabolism is critical in the SSA given that there are over 25 million people living with HIV in the region. Regarding ART and Vitamin D, antiretroviral therapy (ART) is a lifeline, but it comes with metabolic costs. For instance, Efavirenz (EFV), which is an NNRTI that served as the backbone of first-line therapy in SSA for decades, induces the hepatic enzyme CYP24A1, and this enzyme catabolizes 25(OH)D into inactive metabolites, leading to profound VDD (49). Studies have shown that HIV+ patients on EFV have significantly lower Vitamin D levels than those on other regimens (50). Furthermore, Tenofovir (TDF), often used in combination, is associated with secondary hyperparathyroidism and bone loss, further complicating the calcium–Vitamin D axis (49).

In terms of metabolic consequences, lipodystrophy is an important feature, as ART is associated with a redistribution of fat, particularly loss of subcutaneous fat (lipoatrophy) and gain of visceral/hepatic fat (lipohypertrophy). This visceral adiposity drives insulin resistance and mimics the MASLD phenotype (49). Additionally, inflammation persists even with viral suppression because HIV is a state of chronic immune activation, and VDD exacerbates this inflammatory state. Consequently, the “Triple Hit” of HIV inflammation, ART toxicity, and traditional obesity puts this population at extreme risk for MASLD and T2DM. A comparison of these unique regional factors against the global context is presented in Table 2.

**Table 2:** The SSA context: Key differences and implications.

Factor	Global/Western Context	Sub-Saharan African Context	Implication for Research & Care
MASLD Genetic Risk (10, 48)	High prevalence of PNPLA3 I148M (risk allele).	Low prevalence of PNPLA3 risk allele (∼19%).	Environmental factors (Diet, VDD) likely have a higher attributable risk fraction in SSA.
Vitamin D Status (19, 21, 45)	Deficiency defined as <20/30 ng/mL; linked to bone risk.	"Vitamin D Paradox": Low total levels but good bone health.	Standard cut-offs may overestimate deficiency; need Free 25(OH)D measurements.
DBP Genetics (19, 21, 45)	Gc1s/Gc2 alleles dominant.	Gc1f allele dominant (African ancestry).	May affect Vitamin D bioavailability and transport efficiency.
Comorbidities (20, 49)	Obesity, Dyslipidaemia, Hypertension.	HIV (high prevalence), ART toxicity (Efavirenz), TB.	HIV/ART status must be stratified in all metabolic studies/trials.
Dietary Driver (7, 9)	High Fructose Corn Syrup, Saturated Fat.	"Nutrition Transition": Rapid shift to refined carbs & seed oils.	Fortification of staples (oil/flour) is a key intervention point.
T2DM Phenotype (42, 43)	Strongly associated with high BMI (General Obesity).	Often presents at lower BMI (“Lean Diabetes”).	Visceral and Ectopic fat accumulation is more critical than BMI.
Early Life Programming (51, 52)	Focus on childhood obesity prevention.	High prevalence of maternal undernutrition & “Thrifty Phenotype.”	Maternal Vitamin D supplementation may be a critical long-term NCD prevention strategy.

To systematically visualize the disparities in current literature and identify high-priority areas for future investigation, the contrast between established global evidence and the existing voids in the Sub-Saharan African context is summarized in Table 3.

**Table 3:** Research gaps in global vs. Sub-Saharan African evidence.

Research Domain	Global/Western Evidence	Sub-Saharan Africa Evidence	Gap Severity
MASLD Epidemiology	Robust: Prevalence and risk factors well-defined across large cohorts (1, 5, 22).	Emerging: Limited to hospital-based studies; population-level data is sparse (9, 39).	Moderate
Genetic Drivers (PNPLA3)	Established: PNPLA3 I148M is the dominant risk variant (47).	Paradoxical: Risk allele is rare; alternative genetic drivers undefined (10, 48).	High
Vitamin D Status Assessment	Standardized: Defined cut-offs (<20/30 ng/mL) based on bone health (15).	Controversial: “Vitamin D Paradox” complicates definition; lack of “Free Vit D” data (18, 21, 45).	High
Mechanistic Pathways	Advanced: VDR role in insulin signaling and inflammation well-mapped (12, 30, 31).	Absent: No direct mechanistic studies on African-specific cell lines or tissues.	Major gap
Clinical Trials (Efficacy)	Inconsistent: Mixed results from numerous RCTs (13, 14, 32).	Non-Existent: No RCTs specifically testing Vit D for MASLD/T2DM in SSA (51).	Major gap
HIV/ART Interaction	Low Priority: HIV is a managed chronic condition with lower population burden.	Critical: High burden; Efavirenz toxicity & lipodystrophy are major metabolic drivers (20, 51, 61).	High
Early Life Programming (DOHaD)	Growing: Focus on childhood obesity prevention (53).	Urgent: High rates of maternal undernutrition & “thrifty phenotype” programming (52).	High

### 3.4 Emerging perspectives and hypotheses

Synthesizing the global biology with the regional reality allows us to formulate specific hypotheses and emerging perspectives for SSA.

#### 3.4.1 The gut-liver-Vitamin D axis in SSA

The gut microbiome is increasingly recognized as a metabolic organ, and Vitamin D plays a crucial role in maintaining gut barrier integrity (tight junction proteins) while also modulating microbiome composition by promoting beneficial phyla like *Akkermansia* and reducing *Firmicutes* (35). In this context, the “Nutrition Transition” in SSA is driving a specific form of dysbiosis, as traditional diets rich in resistant starch and fibre are being replaced by low-fibre, high-sugar processed foods. Consequently, this dietary shift, combined with widespread VDD, may compromise the intestinal barrier.

As a result, a “leaky gut” allows the translocation of bacterial endotoxins (Lipopolysaccharide — LPS) into the portal circulation, leading to what is described as “Metabolic Endotoxemia.” These endotoxins then travel directly to the liver, where they activate TLR4 on Kupffer cells, thereby triggering the inflammatory cascade that drives MASH and systemic insulin resistance (53, 54). Moreover, emerging evidence suggests that Vitamin D supplementation in SSA could potentially “seal” the gut barrier and reduce the endotoxic load on the liver, a mechanism that might be particularly effective in populations with high infectious burdens (36).

#### 3.4.2 The barker hypothesis: developmental origins of metabolic disease

The “Barker Hypothesis” or DOHaD suggests that the intrauterine environment programs the fetus's metabolic set-points for life, and this framework is particularly relevant in the SSA scenario, where a significant proportion of the current adult population was born during periods of economic hardship, famine, or maternal undernutrition, meaning they were born with “thrifty” phenotypes programmed to conserve energy (55). As these individuals now live in an obesogenic urban environment, the resulting “mismatch” between early-life programming and current lifestyle dramatically amplifies metabolic risk. Within this context, Vitamin D's role becomes important, as recent evidence links maternal VDD to epigenetic modifications (DNA methylation) in offspring that predispose them to adiposity and metabolic dysfunction (51, 52). Consequently, the high prevalence of VDD in pregnant women in SSA today may be programming the next generation for an even more severe wave of MASLD and T2DM, and addressing maternal Vitamin D status is thus not just an obstetric concern but may be a long-term NCD prevention strategy (56).

#### 3.4.3 Fortification and the food environment

The efficacy of Vitamin D as a public health tool depends on delivery systems, and in terms of the fortification landscape, many SSA countries, including Nigeria and South Africa, have mandatory food fortification mandates for staples like wheat flour, maize meal, and cooking oil (57). However, these initiatives typically focus on Vitamin A, Iron, and Zinc, whereas Vitamin D fortification is often voluntary or absent (58). Moreover, even where standards exist, such as Kenya's requirement for Vitamin A in fats and oils, implementation gaps are evident because compliance is variable (59). Furthermore, the stability of vitamins in cooking oils used for high-heat frying, which is common in West African cuisine, is questionable. In addition, the so-called “oil paradox” presents another challenge, as the transition to cheap vegetable oils, often rich in omega-6 PUFAs, may be promoting inflammation, and if these oils are not fortified with Vitamin D, which has anti-inflammatory properties, the dietary shift is doubly harmful. A comprehensive synthesis of the key claims, evidence strength, and supporting literature identified in this review is detailed in Table 4.

**Table 4:** Key findings and supporting evidence identified in the literature.

Findings	Evidence Strength	Rationale	Studies (Ref)
Vitamin D deficiency is associated with increased risk/severity of MASLD and T2DM	Strong	Supported by umbrella reviews, meta-analyses, and large observational studies linking deficiency to insulin resistance and liver fat.	(13, 15, 23, 28, 60)
Vitamin D exerts metabolic protection via the VDR axis (insulin sensitivity, anti-inflammatory)	Strong	Robust mechanistic evidence from molecular and cellular studies demonstrating VDR's role in lipid metabolism and immune modulation.	(11, 12, 24, 30, 31, 54)
Clinical trials of Vitamin D supplementation yield inconsistent results	Moderate	RCTs show mixed outcomes; benefits often limited to specific subgroups, obscured by heterogeneity in dosing and population.	(14, 27, 32, 37, 38)
There is a critical data gap regarding Vitamin D and MASLD in Sub-Saharan Africa	Strong	Systematic reviews confirm a lack of region-specific data despite high prevalence of risk factors (urbanization, infectious disease).	(1, 4, 9, 16, 60)
MASLD in SSA is distinct due to “Triple Burden” (Diet, Urbanization, HIV)	Moderate	Emerging evidence links rapid nutrition transition and HIV/ART to unique metabolic phenotypes, distinct from Western cohorts.	(2, 7–9, 20)
Genetic factors (PNPLA3, DBP) alter the risk profile in African populations	Strong	Genetic studies consistently show low PNPLA3 risk allele frequency in Africans, suggesting environmental drivers (like VDD) are more dominant.	(10, 19, 47, 48)
Vitamin D fortification is a plausible public health strategy for SSA	Moderate	Policy assessments suggest feasibility of fortification (e.g., cooking oil), though efficacy trials for metabolic outcomes are pending.	(57–59)

### 3.5 Future research directions

The current evidence base allows the construction of a robust conceptual framework, but empirical data is needed to validate it. The following targeted research directions for SSA are proposed.

#### 3.5.1 Defining ancestry-specific reference ranges

There is need to move beyond the “one size fits all” definition of VDD. Large-scale cross-sectional studies in diverse African ethnic groups (e.g., Bantu, Nilotic, Khoisan) are required, measuring total 25(OH)D and Free 25(OH)D (directly measured), DBP and VDR genotypes, and markers of calcium homeostasis (PTH, Calcium). In this way, the goal is to establish “physiological” cut-offs for Vitamin D that correlate with metabolic risk (HOMA-IR, Liver Fat) rather than just bone health (21).

#### 3.5.2 The “SSA-metabolic” randomized controlled trial

Global RCTs have been inconclusive, and there is need an SSA-specific trial design. This will involve a multi-center RCT (e.g., Nigeria, Kenya, South Africa) enrolling pre-diabetic patients with ultrasound-diagnosed MASLD and comparing high-dose Vitamin D supplementation with placebo. Moreover, participants must be stratified by baseline Vitamin D status (Deficient vs. Insufficient), HIV Status (On ART vs. Negative), and genotype (VDR variants), while the endpoints should include change in Liver Fat Content (via MRI-PDFF or FibroScan CAP), Insulin Sensitivity (HOMA-IR), and inflammatory markers (hs-CRP, Cytokines).

#### 3.5.3 Investigating the viral-metabolic interface

Given the high prevalence of HIV, specific studies are needed to disentangle the effects of the virus, the drugs, and the vitamin, leading to the key question: Can Vitamin D supplementation mitigate the metabolic toxicity of Efavirenz or Dolutegravir-based ART regimens, and does it reduce the accumulation of visceral/hepatic fat in this specific population (61)?

#### 3.5.4 Longitudinal “life-course” studies

Finally, leveraging existing birth cohorts (like the Drakenstein study in South Africa) is essential to track the long-term metabolic outcomes of maternal Vitamin D status, and the goal is to test the DOHaD hypothesis: Does maternal VDD predict liver fat accumulation in the African child/adolescent? (62).

## 4 Discussion

The evidence from this study supports a strong association between vitamin D deficiency and increased risk or severity of MASLD and T2DM, mediated by effects on insulin sensitivity, inflammation, and hepatic lipid metabolism (13, 15, 23, 28, 60), and mechanistic studies provide a plausible biological basis for these associations, implicating the vitamin D–VDR axis in key metabolic and immune pathways (12, 24, 30, 31, 54). However, clinical trials of vitamin D supplementation yield inconsistent results, with benefits often limited to specific subgroups or mild disease stages (27, 32, 37, 38), and methodological heterogeneity, population differences, and variable dosing regimens contribute to these discrepancies (14, 29). Consequently, the lack of robust data from Sub-Saharan Africa is a significant gap, given the region's unique risk factors and rapidly rising burden of MASLD and T2DM (4, 9, 16, 60), and addressing vitamin D deficiency may offer a low-cost, adjunctive strategy for metabolic disease prevention, although region-specific research is urgently needed to inform public health policies (1, 25).

In light of this, the “MASLD Spectrum” in Sub-Saharan Africa is emerging as a formidable public health challenge, distinct in its epidemiology and genetics from the disease described in Western literature, as it is a condition shaped by the collision of rapid urbanization, dietary transition, and a persistent burden of infectious disease (2, 7–9, 20). This scoping review positions Vitamin D at the center of this complex web, and the global evidence provides a compelling biological rationale: Vitamin D is a potent guardian of metabolic health, essential for insulin secretion, sensitivity, and the suppression of hepatic inflammation (11, 31, 33). Moreover, in SSA, the widespread prevalence of Vitamin D deficiency, driven by urbanization and potentially misunderstood due to genetic variations in binding proteins, represents a massive, unaddressed risk factor (16, 19, 21, 45).

Therefore, the “Specific Gap” identified by this review is not merely academic but has profound implications, because if Vitamin D deficiency is a significant modifiable risk factor in the MASLD–T2DM transition in Africans, potentially filling the risk void left by the absence of PNPLA3 (10, 47, 48), then Vitamin D supplementation could represent a potential low-cost, scalable, and safe intervention that could be integrated into public health programs (for instance, via mandatory fortification of cooking oils) (57–59). However, realizing this potential requires us to shed Eurocentric assumptions about “normal” levels and disease mechanisms, and future research must embrace the complexity of the African context, accounting for genes, germs (HIV), and geography (43, 46, 49, 61). Only by rigorously investigating these SSA-specific dimensions can we turn the promise of Vitamin D into a tangible tool to halt the rising tide of cardiometabolic disease in the region.

## 5 Conclusion

This scoping-narrative review synthesizes a compelling body of evidence positioning Vitamin D as a critical pleiotropic hormone governing the intersection of MASLD and T2DM through established anti-inflammatory, anti-fibrotic, and insulin-sensitizing pathways. Evidence from global clinical trials have yielded inconsistent results and the implications for SSA are distinct and urgent. In a region where the dominant genetic driver of hepatic steatosis (PNPLA3) is rare, widespread VDD, exacerbated by rapid urbanization, dietary transition, and infectious comorbidities, is posited as a plausible environmental driver for the rising metabolic crisis. Consequently, this hypothesis-generating review suggests that resolving the “Vitamin D Paradox” through ancestry-specific reference ranges and validating targeted supplementation strategies represents a high-priority public health frontier, though robust randomized controlled trials are required to confirm efficacy before clinical implementation.

## Data Availability

The original contributions presented in the study are included in the article/Supplementary Material, further inquiries can be directed to the corresponding author.

## References

[1] Chan WK Chuah KH Rajaram RB Lim LL Ratnasingam J Vethakkan SR. Metabolic dysfunction-associated steatotic liver disease (MASLD): a state-of-the-art review. J Obes Metab Syndr. (2023) 32(3):193–206. 10.7570/jomes23052 PMC10583766 37700494

[2] Lorek D Łupina K Bisaga W Malicki D Stępień W Kumor L The socioeconomic and environmental determinants of metabolic dysfunction-associated steatotic liver disease: understanding inequalities in prevalence and outcomes. Korean J Fam Med. (2025) 46:61–9. 10.4082/kjfm.25.0027 40139924 PMC11969182

[3] Basil B Myke-Mbata BK Eze OE Akubue AU. From adiposity to steatosis: metabolic dysfunction-associated steatotic liver disease, a hepatic expression of metabolic syndrome—current insights and future directions. Clin Diabetes Endocrinol. (2024) 10(1):39. 10.1186/s40842-024-00187-4 39617908 PMC11610122

[4] Motala AA Mbanya JC Ramaiya K Pirie FJ Ekoru K. Type 2 diabetes mellitus in sub-Saharan Africa: challenges and opportunities. Nat Rev Endocrinol. (2022) 18(4):219–29. 10.1038/s41574-021-00613-y 34983969

[5] Gancheva S Roden M Castera L. Diabetes as a risk factor for MASH progression. Diabetes Res Clin Pract. (2024) 217:111846. 10.1016/j.diabres.2024.111846 39245423

[6] London A Lundsgaard AM Kiens B Bojsen-Møller KN. The role of hepatic fat accumulation in glucose and insulin homeostasis—dysregulation by the liver. J Clin Med. (2021) 10(3):390. 10.3390/jcm10030390 33498493 PMC7864173

[7] Nel JH Steyn NP. The nutrition transition and the double burden of malnutrition in Sub-Saharan African countries: how do these countries compare with the recommended LANCET COMMISSION global diet? Int J Environ Res Public Health. (2022) 19(24):16791. 10.3390/ijerph192416791 36554669 PMC9779835

[8] Popkin BM Corvalan C Grummer-Strawn LM. Dynamics of the double burden of malnutrition and the changing nutrition reality. Lancet. (2020) 395(10217):65–74. 10.1016/S0140-6736(19)32497-3 31852602 PMC7179702

[9] Sindato EM Kajogoo VD Ngajilo G Degu WA Khan Z Mlawa G. Prevalence and risk factors of metabolic-associated fatty liver disease in sub-Saharan Africa: a systematic review and meta-analysis. Front Gastroenterol. (2025) 4:1506032. 10.3389/fgstr.2025.1506032 PMC12952399 41822265

[10] Kozlitina J Sookoian S. Global epidemiological impact of PNPLA3 I148M on liver disease. Liver Int. (2025) 45(3):e16123. 10.1111/liv.16123 39373119 PMC11815610

[11] Fuentes-Barría H Aguilera-Eguía R Flores-Fernández C Angarita-Davila L Rojas-Gómez D Alarcón-Rivera M Vitamin D and type 2 diabetes mellitus: molecular mechanisms and clinical implications—a narrative review. Int J Mol Sci. (2025) 26(5):2153. 10.3390/ijms26052153 40076782 PMC11900948

[12] Aggeletopoulou I Tsounis EP Triantos C. Vitamin D and metabolic dysfunction-associated steatotic liver disease (MASLD): novel mechanistic insights. Int J Mol Sci. (2024) 25(9):4901. 10.3390/ijms25094901 38732118 PMC11084591

[13] Cheng L Lv C Xue L Zhang C Wang L Wang X The prevention and improvement effects of Vitamin D on type 2 diabetes mellitus: evidence from an umbrella review on meta-analyses of cohort studies and randomized controlled trials. Front Nutr. (2024) 11:1462535. 10.3389/fnut.2024.1462535 39525505 PMC11543531

[14] Martinekova P Obeidat M Topala M Váncsa S Veres DS Zolcsák Á Role of Vitamin D supplementation in chronic liver disease: a systematic review and meta-analysis of randomized controlled trials. Nutr Rev. (2025) 83(11):2043–54. 10.1093/nutrit/nuaf117 40644459 PMC12512233

[15] Zhao S Qian F Wan Z Chen X Pan A Liu G. Vitamin D and major chronic diseases. Trends Endocrinol Metab. (2024) 35:1050–61. 10.1016/j.tem.2024.04.018 38824035

[16] Mogire RM Mutua A Kimita W Kamau A Bejon P Pettifor JM Prevalence of Vitamin D deficiency in Africa: a systematic review and meta-analysis. Lancet Glob Health. (2020) 8(1):e134–142. 10.1016/S2214-109X(19)30457-7 31786117 PMC7024961

[17] Mangal DK Shaikh N Tolani H Gautam D Pandey AK Sonnathi Y Burden of micronutrient deficiency among patients with type 2 diabetes: systematic review and meta-analysis. BMJ Nutr Prev Health. (2025) 8(1):e000950. 10.1136/bmjnph-2024-000950 40771523 PMC12322531

[18] Basil B Adiri W Ugwu LE Okoro NI. Association between Vitamin D and metabolic-associated steatotic liver disease in type 2 diabetes mellitus patients: a systematic review and meta-analysis. Diabetol Metab Syndr. (2025) 17(1):457. 10.1186/s13098-025-02031-w 41444644 PMC12729236

[19] Aloia J Mikhail M Dhaliwal R Shieh A Usera G Stolberg A Free 25(OH)D and the Vitamin D paradox in African Americans. J Clin Endocrinol Metab. (2015) 100(11):4124–31. 10.1210/JC.2015-2066 26161453 PMC4570168

[20] Asgedom YS Kebede TM Gebrekidan AY Koyira MM Azeze GA Lombebo AA Prevalence of metabolic syndrome among people living with human immunodeficiency virus in sub-Saharan Africa: a systematic review and meta-analysis. Sci Rep. (2024) 14(1):11709. 10.1038/s41598-024-62497-y 38777850 PMC11111734

[21] Nielson CM Jones KS Chun RF Jacobs JM Wang Y Hewison M Free 25-hydroxyvitamin D: impact of Vitamin D binding protein assays on racial-genotypic associations. J Clin Endocrinol Metab. (2016) 101(11):4317–25. 10.1210/jc.2016-1104 PMC4870848 27007693

[22] Peters MDJ Marnie C Colquhoun H Garritty CM Hempel S Horsley T Scoping reviews: reinforcing and advancing the methodology and application. Syst Rev. (2021) 10(1):263. 10.1186/s13643-021-01821-3 34625095 PMC8499488

[23] Barchetta I Cimini FA Cavallo MG. Vitamin D and metabolic dysfunction-associated fatty liver disease (MAFLD): an update. Nutrients. (2020) 12(11):3302. 10.3390/nu12113302 33126575 PMC7693133

[24] Pop T Sîrbe C Bența G Mititelu A Grama A. The role of Vitamin D and Vitamin D binding protein in chronic liver diseases. Int J Mol Sci. (2022) 23(18):10705. 10.3390/ijms231810705 36142636 PMC9503777

[25] Vrentzos E Pavlidis G Korakas E Kountouri A Pliouta L Dimitriadis G Nutraceutical strategies for metabolic dysfunction-associated steatotic liver disease (MASLD): a path to liver health. Nutrients. (2025) 17(10):1657. 10.3390/nu17101657 40431398 PMC12113997

[26] Mousa A Naderpoor N Johnson J Sourris K De Courten MPJ Wilson K Effect of Vitamin D supplementation on inflammation and nuclear factor kappa-B activity in overweight/obese adults: a randomized placebo-controlled trial. Sci Rep. (2017) 7(1):14868. 10.1038/s41598-017-15264-1 29123173 PMC5680306

[27] Dashti F Mousavi SM Larijani B Esmaillzadeh A. The effects of Vitamin D supplementation on inflammatory biomarkers in patients with abnormal glucose homeostasis: a systematic review and meta-analysis of randomized controlled trials. Pharmacol Res. (2021) 170:105727. 10.1016/j.phrs.2021.105727 34126229

[28] Xing Y Cheng T Zhou F Ma H. The association between Vitamin D and type 2 diabetes mellitus complicated with non-alcoholic fatty liver disease: an observational cross-sectional study. Diabetes Metab Syndr Obes. (2022) 15:269–80. 10.2147/DMSO.S348870 35140487 PMC8819170

[29] Mattioli AV Coppi F Severino P Penna C Pagliaro P Dei Cas A A personalized approach to Vitamin D supplementation in cardiovascular health beyond the bone: an expert consensus by the Italian national institute for cardiovascular research. Nutrients. (2024) 17(1):115. 10.3390/nu17010115 39796548 PMC11722835

[30] Yaribeygi H Maleki M Sathyapalan T Iranpanah H Orafai H Jamialahmadi T The molecular mechanisms by which Vitamin D improve glucose homeostasis: a mechanistic review. Life Sci. (2020) 259:117305. 10.1016/j.lfs.2020.117305 31953161

[31] Wu J Atkins A Downes M Wei Z. Vitamin D in diabetes: uncovering the sunshine hormone’s role in glucose metabolism and beyond. Nutrients. (2023) 15(8):1997. 10.3390/nu15081997 37111216 PMC10142687

[32] Qi KJ Zhao ZT Zhang W Yang F. The impacts of Vitamin D supplementation in adults with metabolic syndrome: a systematic review and meta-analysis of randomized controlled trials. Front Pharmacol. (2022) 13:1033026. 10.3389/fphar.2022.1033026 36278155 PMC9581173

[33] Szymczak-Pajor I Miazek K Selmi A Balcerczyk A Śliwińska A. The action of Vitamin D in adipose tissue: is there the link between Vitamin D deficiency and adipose tissue-related metabolic disorders? Int J Mol Sci. (2022) 23(2):956. 10.3390/ijms23020956 35055140 PMC8779075

[34] Park CY Shin S Han SN. Multifaceted roles of Vitamin D for diabetes: from immunomodulatory functions to metabolic regulations. Nutrients. (2024) 16(18):3185. 10.3390/nu16183185 39339785 PMC11435169

[35] Sukik A Alalwani J Ganji V. Vitamin D, gut microbiota, and cardiometabolic diseases—a possible three-way axis. Int J Mol Sci. (2023) 24(2):940. 10.3390/ijms24020940 36674452 PMC9866669

[36] Charoenngam N Shirvani A Kalajian TA Song A Holick MF. The effect of various doses of oral Vitamin D(3) supplementation on gut microbiota in healthy adults: a randomized, double-blinded, dose-response study. Anticancer Res. (2020) 40(1):551–6. 10.21873/anticanres.13984 31892611

[37] Vasdeki D Tsamos G Dimakakos E Patriarcheas V Koufakis T Kotsa K Vitamin D supplementation: shedding light on the role of the sunshine vitamin in the prevention and management of type 2 diabetes and its complications. Nutrients. (2024) 16(21):3651. 10.3390/nu16213651 39519484 PMC11547801

[38] Sacerdote A Dave P Lokshin V Bahtiyar G. Type 2 diabetes mellitus, insulin resistance, and Vitamin D. Curr Diab Rep. (2019) 19(10):101. 10.1007/s11892-019-1201-y 31506836

[39] Anyanwu A Olopade O Onung S Odeniyi I Coker H Fasanmade O Serum Vitamin D levels in persons with type 2 diabetes mellitus in Lagos, Nigeria. Int J Diabetes Clin Res. (2020) 7(1):133. 10.23937/2377-3634/1410133

[40] Karau PB Kirna B Amayo E Joshi M Ngare S Muriira G. The prevalence of Vitamin D deficiency among patients with type 2 diabetes seen at a referral hospital in Kenya. Pan Afr Med J. (2019) 34:38. 10.11604/pamj.2019.34.38.18936 31762905 PMC6859033

[41] Todowede OO Mianda SZ Sartorius B. Prevalence of metabolic syndrome among HIV-positive and HIV-negative populations in sub-Saharan Africa—a systematic review and meta-analysis. Syst Rev. (2019) 8(1):4. 10.1186/s13643-018-0927-y 30606249 PMC6317235

[42] Goedecke JH Mendham AE. Pathophysiology of type 2 diabetes in sub-Saharan Africans. Diabetologia. (2022) 65(12):1967–80. 10.1007/s00125-022-05795-2 36166072 PMC9630207

[43] Masemola M Mendham AE Micklesfield LK Pheiffer C Hawley J Kengne AP Regional adiposity and insulin sensitivity-interactions with menopause and HIV in middle-aged black African women. J Clin Endocrinol Metab. (2024) 110(1):16–29. 10.1210/clinem/dgae447 38950129

[44] Brown LL Cohen B Tabor D Zappalà G Maruvada P Coates PM. The Vitamin D paradox in black Americans: a systems-based approach to investigating clinical practice, research, and public health—expert panel meeting report. BMC Proc. (2018) 12(Suppl 6):6. 10.1186/s12919-018-0102-4 30044889 PMC5954269

[45] Powe CE Evans MK Wenger J Zonderman AB Berg AH Nalls M Vitamin D–binding protein and Vitamin D status of black Americans and white Americans. N Engl J Med. (2013) 369(21):1991–2000. 10.1056/nejmoa1306357 24256378 PMC4030388

[46] Mishra DK Wu Y Sarkissyan M Sarkissyan S Chen Z Shang X Vitamin D receptor gene polymorphisms and prognosis of breast cancer among African-American and hispanic women. PLoS One. (2013) 8(3):e57967. 10.1371/journal.pone.0057967 23554871 PMC3595235

[47] Wagenknecht LE Palmer ND Bowden DW Rotter JI Norris JM Ziegler J Association of PNPLA3 with non-alcoholic fatty liver disease in a minority cohort: the insulin resistance atherosclerosis family study. Liver Int. (2011) 31(3):412–6. 10.1111/j.1478-3231.2010.02444.x 21281435 PMC3703938

[48] Cavalcante LN Porto J Mazo D Longatto-Filho A Stefano JT Lyra AC African genetic ancestry is associated with lower frequency of PNPLA3 G allele in non-alcoholic fatty liver in an admixed population. Ann Hepatol. (2022) 27(5):100728. 10.1016/j.aohep.2022.100728 35710086

[49] Ashenafi S Amogne W Kassa E Gebreselassie N Bekele A Aseffa G Daily nutritional supplementation with Vitamin d3 and phenylbutyrate to treatment-naïve HIV patients tested in a randomized placebo-controlled trial. Nutrients. (2019) 11(1):133. 10.3390/nu11010133 30634590 PMC6356462

[50] Dave JA Cohen K Micklesfield LK Maartens G Levitt NS. Antiretroviral therapy, especially efavirenz, is associated with low bone mineral density in HIV-infected South Africans. PLoS One. (2015) 10(12):e0144286. 10.1371/journal.pone.0144286 26633015 PMC4669137

[51] Ideraabdullah FY Belenchia AM Rosenfeld CS Kullman SW Knuth M Mahapatra D Maternal Vitamin D deficiency and developmental origins of health and disease (DOHaD). J Endocrinol. (2019) 241(1):R1–13. 10.1530/JOE-18-0541 30909167 PMC6717694

[52] Wu Y Zeng Y Zhang Q Xiao X. The role of maternal Vitamin D deficiency in offspring obesity: a narrative review. Nutrients. (2023) 15(3):533. 10.3390/nu15030533 36771240 PMC9919568

[53] An L Wirth U Koch D Schirren M Drefs M Koliogiannis D The role of gut-derived lipopolysaccharides and the intestinal barrier in fatty liver diseases. J Gastrointest Surg. (2022) 26(4):795–806. 10.1007/s11605-021-05188-7 PMC8926958 34734369

[54] Xu GX Wei S Yu C Zhao SQ Yang WJ Feng YH Activation of Kupffer cells in NAFLD and NASH: mechanisms and therapeutic interventions. Front Cell Dev Biol. (2023) 11:1199519. 10.3389/fcell.2023.1199519 37261074 PMC10228659

[55] Chandra M. Developmental origins of non-communicable chronic diseases: role of fetal undernutrition and gut dysbiosis in infancy. Children. (2024) 11(11):1387. 10.3390/children11111387 39594962 PMC11592819

[56] Ramírez-Alarcón K Sánchez-Agurto Á Lamperti L Martorell M. Epigenetics, maternal diet and metabolic programming. Open Biol J. (2019) 7:45–56. 10.2174/1874196701907010045

[57] Aaron GJ Friesen VM Jungjohann S Garrett GS Neufeld LM Myatt M. Coverage of large-scale food fortification of edible oil, wheat flour, and maize flour varies greatly by vehicle and country but is consistently lower among the most vulnerable: results from coverage surveys in 8 countries. J Nutr. (2017) 147(6):1222S–32. 10.3945/jn.116.245753 PMC5404213 28404836

[58] Coomson JB Smith NW McNabb W. Contribution of large-scale food fortification to micronutrient requirements of women of reproductive age in Sub-Saharan Africa. Proc Nutr Soc. (2025) 84:E168. 10.1017/S0029665125100244

[59] Theriault V Kirimi L Wineman A Kinyumu E Tschirley D. Assessment of the policy enabling environment for large-scale food fortification: a novel framework with an application to Kenya. PLOS Glob Public Health. (2024) 4(5):e0003211. 10.1371/journal.pgph.0003211 38753812 PMC11098474

[60] Muhihi A Fawzi WW Aboud S Nagu TJ Ulenga N Wang M Cholecalciferol supplementation does not affect the risk of HIV progression, viral suppression, comorbidities, weight loss, and depression among tanzanian adults initiating antiretroviral therapy: secondary outcomes of a randomized trial. J Nutr. (2022) 152(8):1983–90. 10.1093/jn/nxac096 35460249 PMC9361733

[61] Ncayiyana JR Martinez L Goddard E Myer L Zar HJ. Prevalence and correlates of Vitamin D deficiency among young South African infants: a birth cohort study. Nutrients. (2021) 13(5):1500. 10.3390/nu13051500 33946851 PMC8146842

[62] Chen L Wagner CL Dong Y Wang X Shary JR Huang Y Effects of maternal Vitamin D3 supplementation on offspring epigenetic clock of gestational age at birth: a *post-hoc* analysis of a randomized controlled trial. Epigenetics. (2020) 15(11):1254–69. 10.1080/15592294.2020.1734148 PMC7518698 32089064

